# Prenatal maternal bereavement and risk of eating disorders in infants and toddlers: a population-based cohort study

**DOI:** 10.1186/s12888-015-0612-9

**Published:** 2015-09-24

**Authors:** Xiujuan Su, Beibei Xu, Hong Liang, Jørn Olsen, Wei Yuan, Sven Cnattingius, Krisztina D. László, Jiong Li

**Affiliations:** 1 Department of Women’s and Children’s Health Care, Shanghai First Maternity and Infant Hospital, Tongji University School of Medicine, Shanghai, 200040 PR China; 2 Section for Epidemiology, Department of Public Health, Aarhus University, Aarhus, 8000 Denmark; 3 Medical Informatics Center, Peking University, Beijing, PR China; 4 Department of Reproductive Epidemiology and Social Science, National Population and Family Planning Key Laboratory of Contraceptive Drugs and Devices, Shanghai Institute of Planned Parenthood Research, Shanghai, 200032 PR China; 5 Clinical Epidemiology Unit, Department of Medicine, Karolinska University Hospital and Karolinska Institute, Stockholm, Sweden

**Keywords:** Bereavement, Eating disorders, Hypothalamus-pituitary-adrenal axis, Prenatal stress

## Abstract

**Background:**

Prenatal stress has been associated to a number of neuropsychiatric diseases but its role on the development of eating disorders (ED) remains unknown. Infants and toddlers with feeding or eating disorders are also at an increased risk of such diseases in later childhood and adolescence. We aimed to examine whether prenatal stress following maternal bereavement is associated with ED in infants and toddlers.

**Methods:**

This population-based cohort study included children born from 1977 to 2008 in Denmark (N = 2,127,126) and from 1977 to 2006 in Sweden (N = 2,974,908). Children were categorized as exposed if they were born to mothers who lost a close relative one year prior to or during pregnancy and were categorized as unexposed otherwise. They were followed until the age of 3 for a first diagnosis of ED. Poisson regression models were used to examine incidence rate ratio (IRR) between the exposed and the unexposed cohort.

**Results:**

A total of 9,403 ED cases were identified and 179 of whom were in the exposed cohort. Offspring born to mothers bereaved by loss of a core family member (older child or spouse) within the six months before pregnancy had a higher risk of ED than the unexposed offspring (IRR: 1.63, 95 % confidence intervals (CI): 1.07–2.47). In stratified analyses, bereavement during the six months before pregnancy was associated with an increased risk of ED in boys (IRR: 2.21, 95 % CI: 1.28–3.82), but not in girls (IRR: 1.18, 95 % CI: 0.61–2.27).

**Discussion:**

This is the first population-based study to explore the association between prenatal stress and the risk of ED in infants and toddlers within two Nordic countries. This study added new evidence of early life stress for etiology of ED while the potential mechanism still needs further studies.

**Conclusions:**

Prenatal stress following maternal bereavement by loss of a core family member is associated with an increased risk of ED among infants and toddlers. The six months before conception may be a susceptible time window, especially for boys.

## Background

The lifetime prevalence of all types of eating disorders (ED) is approximately 5 % and they may lead to profound and long-lasting physical and psychosocial morbidity [1]. Infants with feeding problems are at an increased risk of ED in later childhood and adolescence [2]. ED are characterized by food refusal for at least one month, growth deficiency and an apparent lack of appetite in infants and toddlers [3]. Early problems in eating behaviors are reported to be more common in infants with developmental delays (80 %) than in normally developed infants (25 %) [4]. However, despite its important consequences, the etiology of ED in infants and toddlers is poorly understood [5].

Eating behavior is a complex process controlled by the neuroendocrine system, of which the hypothalamus-pituitary-adrenal-axis (HPA axis) is a major component [6, 7]. Dysregulation of the HPA axis is associated with ED [8–11]. ED may have its origins in prenatal life and accumulating evidence from experimental studies suggests that the prenatal stress has a programming effect, particularly on the activity of the HPA axis [12, 13]. Findings from a recent review suggest that prenatal stress exposure, the activity of the HPA axis, and feeding regulation in early years of life are closely linked [14]. However, there has been little empirical evidence on the potential effect of preconceptional and prenatal maternal stress on the risk of ED in infants and toddlers.

Maternal bereavement during or shortly before pregnancy, as an indicator of severe stress, has been associated with a number of psychiatric disorders in the offspring, including autism spectrum disorder (ASD), attention deficit hyperactivity disorder (ADHD) [15, 16], and affective psychosis [17]. The risks are often higher for children bereaved by loss of a core family member (i.e., older child or spouse) than by loss of an extended family member (i.e., mother’s sibling or parent), suggesting the presence of dose-response effect. Findings regarding the importance of the timing of stress have been mixed.

We hypothesized that prenatal stress following maternal bereavement may increase the risk of ED in infants and toddlers. As ED is rarely diagnosed in early ages, we use combined data from nationwide registries in two countries (Denmark and Sweden) to examine the association. The large study allowed us to investigate the dose-response patterns and the importance of the timing of stress observed in other psychiatric disorders. In addition, as sex difference was observed for effect of prenatal stress on some other psychiatric diseases [16, 18], we also investigated whether association between prenatal stress and ED differed by sex.

## Methods

This study was based on secondary data and all analyses were performed on the secure platform of Statistics Denmark using encrypted identification numbers and thus no access to personal identification numbers of the participants. The study was approved by the Danish Data Protection Agency (No. 2008-41-2680) and Scientific Ethics Committee of Central Jutland Region (No. M-201000252) and Karolinska Institutet (No. 2008/4:6).

### Study design and participants

We conducted a population-based cohort study by combining data from several national registries from Denmark and Sweden. The unique personal identification number, assigned in both countries to all legal residents, allows individual record linkage across all national registries.

We used data from the Danish Civil Registry System and the Swedish Multi-generation Register to identify children born from 1977 to 2008 in Denmark (N = 2,136,450) and from 1977 to 2006 in Sweden (N = 2,982,647) [19, 20]. We excluded children with unknown sex (N = 20), with no linkage to the mothers (N = 8,889), diagnosed with ED as a comorbidity to cleft lip and palate or any type of digestive system malformation (N = 153), born to mothers who lost a close relative caused by ED (N = 156), children to mothers with no linkage to any relatives (N = 7,453), with missing or implausible maternal age (unknown, ≤14 or ≥60 years, N = 392). Our study population consisted of 5,102,034 children born in Denmark (N = 2,127,126) and Sweden (N = 2,974,908).

### Exposure and outcome

We categorized children as exposed if their mothers lost an older child, a sibling, or a parent one year prior to or during pregnancy, or if the mother lost a spouse during pregnancy. Only the first time exposure information was used when one case was exposed to maternal bereavement more than once within the exposure window (0.04 %). The remaining children were included in the unexposed cohort.

Information on ED for the index child was obtained from the Danish Psychiatric Central Research Register (N = 5) [21] and the Danish National Patient Register (N = 2002) [22] in Denmark. The Danish Psychiatric Central Research Register contains information on every psychiatric hospital admission since 1969 and onwards, and information on outpatient treatment is included since 1995. Similar information was obtained from the Swedish Patient Register (N = 7,396), which holds information on psychiatric inpatient care since 1973 [23]. Patients with mild to moderate mental disorders are generally diagnosed and treated by the private practice psychiatrists and general practitioners in cooperation, without hospital contact in Denmark, thus are not registered in the Psychiatric Central Research Register. The diagnosis information was based on *the International Classification of Diseases (ICD). The eighth version* (ICD-8) was used from 1977 to 1993 in Denmark and from 1973 to 1986 in Sweden, *the ninth version* (ICD-9) from 1987 to1996 in Sweden, *the tenth version* (ICD-10) from 1994 onwards in Denmark and from 1997 onwards in Sweden. Based on the diagnosis criteria of feeding disorders proposed by Chatoor [24], a child was defined as a case if he/she had a diagnosis of ED or feeding disorders (ICD-8 codes 306.5 (feeding disturbance); ICD-9 codes 307.B (anorexia nervosa), 307.F (bulimia nervosa); ICD-10 codes F50 (eating disorders), F98.2 (feeding disorder of infancy and childhood), F98.3 (Pica of infancy and childhood)) before the age of 3. Cohort members were followed from birth until the first diagnosis of ED or feeding disorders, death, emigration, the day when they reached 3 years of age, or the last follow up date (December 31, 2010 in Denmark and December 31, 2007 in Sweden), whichever came first.

### Potential confounders

Perinatal factors (parity and the number of fetuses in the pregnancy) and children’s sex were retrieved from the Medical Birth Register [25, 26]. Paternal age and maternal characteristics including age, countries of origin, education were obtained from the Danish Integrated Database for Longitudinal Labor Market Research [27], Swedish Medical Birth Register [25] and the Swedish Education Register [28]. Data on family history of psychiatric disorders was retrieved from the Danish Central Psychiatric Register, the Danish National Patient Register and Swedish Patient Register (ICD-8 codes 290–315, ICD-9 codes 290–319 and ICD-10 codes F00-F99) [21–23].

### Statistical analysis

All data management and analyses were performed with the SAS version 9.2 statistical software packages (SAS Institute, Inc., Cary, North Carolina). Poisson regression models were used to estimate incidence rate ratio (IRR) of ED between exposed and unexposed children.

We divided the exposure window in five periods (12–7 months before pregnancy, 6–0 months before pregnancy, the first trimester, the second trimester and the third trimester) to examine whether a potential effect of preconceptional and prenatal stress following maternal bereavement on ED differed across these periods.

To examine a potential dose-response relationship between maternal bereavement and offspring’s ED risk, we categorized exposed children in two ways, which may represent the stress level of bereavement. (1) according to the causes of death: a) death due to unexpected causes (Swedish codes:79590–79599, 79621, E807-E999 (ICD-8); 798, E807-E999 (ICD-9); R95, R96, R98, V01-Y98 (ICD-10); Danish codes: 795, 810–823, 950–959, 800–807, 825–949, 960–999 (ICD-8); R95-R98, V01-V89, X60-X84 (ICD-10)), and b) death due to other causes; and (2) according to the mother’s relationship of the deceased: a) death of a core family member (an older child or a spouse), and b) death of other relatives (a parent or a sibling). We hypothesized that loss of a core family member would have a stronger effect than loss of a parent or a sibling [29]. Similarly, we anticipated a stronger effect in case of unexpected deaths than in case of other deaths [30].

To examine potential sex-differences in prenatal bereavement and risk of ED in offspring, we performed analyses in boys and girls separately.

We adjusted for the following potential confounders: country (Denmark, Sweden), sex (boy, girl), parity (1, 2, ≥3, unknown), maternal age (15–26 years, 27–30 years, 31–59 years), paternal age (15–28 years, 29–33 years, ≥34 years, unknown), maternal countries of origin (Nordic, others, unknown), number of fetuses in the pregnancy (singleton, multiple, unknown), family history of psychiatric disorders (yes, no) and maternal education (≤9 years, 10–14 years, ≥15 years). Calendar year of follow up was included as a time-dependent variable, while the other covariates were included as time-fixed (Model 1). Since adverse birth outcomes (preterm delivery, low birth weight) have been proposed to be risk factors for ED [31, 32], we adjusted additionally for preterm delivery (yes, no) and birth weight (<2500 g, 2500–4000 g, ≥4000 g) (Model 2).

Finally, we restricted the analyses to children without family history of psychiatric disorders to partly disentangle genetic effects from maternal stress effects [33].

## Results

### Baseline characteristics of the cohort

A total of 86,017 (1.69 %) children were born to mothers experiencing bereavement one year prior to or during pregnancy. The baseline characteristics of the study populations in Denmark and Sweden are presented in Table 1. In both countries, exposed children were more likely to be born to older parents and to have a higher parity than unexposed children.

**Table 1:** Baseline characteristics of the study population

	Denmark			Sweden		
Characteristics	Exposed	Unexposed	*P* value	Exposed	Unexposed	*P* value
	(N = 29,475)	(N = 2,097,651)		(N = 56,542)	(N = 2,918,366)	
	N (%)	N (%)		N (%)	N (%)	
Sex						
Boy	14,981(51)	1,076,217(51)		29,006(51)	1,499,551(51)	
Girl	14,494(49)	1,021,434(49)	0.10	27,536(49)	1,418,815(49)	0.69
Maternal age (years)						
15–26	7,924(27)	708,240(34)		15,422(27)	1,073,318(37)	
27–30	8,613(29)	640,409(31)		15,617(28)	849,158(29)	
31–59	12,938(44)	749,002(36)	<0.01	25,503(45)	995,890(34)	<0.01
Paternal age (years)						
15–28	7,393(25)	622,992(30)		15,161(27)	976,972(33)	
29–33	10,191(35)	726,459(35)		18,847(33)	986,054(34)	
≥ 34	11,137(38)	681,103(32)		22,201(39)	935,866(32)	
Unknown	754(3)	67,097(3)	<0.01	333(1)	19,474(1)	<0.01
Parity						
1	11,035(37)	979,756(47)		18,131(32)	1,223,966(42)	
2	11,723(40)	778,384(37)		21,144(37)	1,056,861(36)	
≥ 3	6,711(23)	333,485(16)		17,265(31)	637,471(22)	
Unknown	6(0)	6,026(0)	<0.01	2(0)	68(0)	<0.01
Number of fetuses in the pregnancy						
Singleton	28,099(95)	1,898,839(91)		55,091(97)	2,846,323(98)	
Multiple	953(3)	64,731(3)		1,446(3)	71,866(2)	
Unknown	423(1)	134,081(6)	<0.01	5(0)	177(0)	0.25
Maternal countries of origin						
Nordic	29,275(99)	2,074,856(99)		54,543(96)	2,603,304(89)	
Others	194(1)	13,129(1)		1,963(3)	302,486(10)	
Unknown	6(0)	9,666(0)	<0.01	36(0)	12,576(0)	<0.01
Family history of psychiatric disorders						
No	26,173(89)	1,912,542(91)		51,896(92)	2,717,203(93)	
Yes	3,302(11)	185,109(9)	<0.01	4,646(8)	201,163(7)	<0.01
Maternal education						
≤ 9 years	8,662(29)	546,022(26)		10,196(18)	487,899(17)	
10–14 years	12,601(43)	825,834(39)		36,664(65)	1,823,765(62)	
≥ 15 years	6,821(23)	438,799(21)		8,620(15)	403,798(14)	
Unknown	1,391(5)	286,996(14)	<0.01	1,062(2)	202,904(7)	<0.01

### Timing-specific and dose-response pattern of prenatal stress (maternal bereavement)

Of 9,403 infants and toddlers with a diagnosis of ED, 179 were exposed to preconceptional and prenatal maternal bereavement. We did not find an association between preconceptional and prenatal maternal bereavement and the overall risk of ED, neither when taking possible confounders into account (Table 2, Model 1), nor when also accounting for possible mediators (Table 2, Model 2). Neither time of exposure (12–7 months before pregnancy, 6–0 months before pregnancy, the first trimester, the second trimester and the third trimester), nor cause of death (unexpected causes and other causes) influenced risk of ED (Table 3).

**Tables 2:** Relative risk of eating disorders and prenatal maternal bereavement according to timing of exposure

Exposure category	Cases	Incidence rate (1/100,000)	Crude IRR	Adjusted IRR (95 % CI)^Model 1^	Adjusted IRR (95 % CI)^Model 2^
Unexposed	9,225	63.00	1.00(ref)	1.00(ref)	1.00(ref)
Any exposed	179	71.32	1.13	1.12(0.97–1.30)	1.10(0.95–1.28)
12–7 months	53	73.12	1.17	1.17(0.90–1.53)	1.14(0.87–1.49)
6–0 months	61	76.82	1.20	1.20(0.93–1.54)	1.18(0.91–1.50)
Prenatal	65	65.60	1.04	1.02(0.80–1.30)	1.02(0.80–1.30)
1st trimester	23	79.07	1.24	1.21(0.80–1.82)	1.19(0.79–1.79)
2nd trimester	26	64.15	1.04	1.01(0.70–1.48)	1.00(0.68–1.45)
3rd trimester	16	54.31	0.85	0.85(0.52–1.39)	0.88(0.54–1.44)

Model 1: Adjusted for country, offspring’s sex, parity, number of fetuses in the pregnancy, paternal age, maternal age, maternal countries of origin, family history of psychiatric disorders, maternal education and calendar year of follow up

Model 2: Adjusted for gestational age and birth weight in addition to the variables from Model 1

*IRR* incidence rate ratio, *CI* confidence intervals

**Tables 3:** Relative risk of eating disorders and prenatal maternal bereavement according to cause of death and timing of exposure

Exposure category	Cases	Incidence rate (1/100,000)	Crude IRR	Adjusted IRR (95 % CI)^Model 1^	Adjusted IRR (95 % CI)^Model 2^
Unexposed	9,225	63.00	1.00(ref)	1.00(ref)	1.00(ref)
Unexpected causes	21	71.15	1.17	1.05(0.69–1.59)	1.03(0.68–1.56)
12–7 months	7	82.44	1.43	1.34(0.67–2.69)	1.29(0.67–2.68)
6–0 months	4	41.11	0.62	0.58(0.22–1.54)	0.56(0.21–1.50)
Prenatal	10	88.55	1.33	1.18(0.64–2.20)	1.18(0.64–2.20)
Other causes	158	71.69	1.13	1.13(0.97–1.33)	1.12(0.95–1.31)
12–7 months	46	72.20	1.13	1.14(0.85–1.52)	1.11(0.83–1.49)
6–0 months	57	82.22	1.28	1.30(1.00–1.68)	1.26(0.97–1.63)
Prenatal	55	62.97	1.00	1.00(0.77–1.30)	1.00(0.76–1.29)

Model 1: Adjusted for country, offspring’s sex, parity, number of fetuses in the delivery, paternal age, maternal age, maternal countries of origin, family history of psychiatric disorders, maternal education and calendar year of follow up

Model 2: Adjusted for gestational age and birth weight in addition to the variables from Model 1

*IRR* incidence rate ratio, *CI* confidence intervals

Compared to unexposed children, children exposed to maternal bereavement by loss of a core family member (an older child or spouse) had an increased risk of ED (IRR: 1.53, 95%CI: 1.13–2.08), while children exposed to maternal bereavement by loss of other relatives were not at an increased risk (IRR: 1.02, 95 % CI: 0.86–1.20) (Table 4). When we additionally stratified children exposed to maternal bereavement into three groups by timing of exposure, we only observed that maternal bereavement related to loss of a core family number during six months before conception was associated with an increased risk of ED (IRR: 1.63, 95 % CI: 1.07–2.47) (Table 4).

**Tables 4:** Relative risk of eating disorders and prenatal maternal bereavement according to type of the deceased relative and time of exposure

Exposure category	Cases	Incidence rate (1/100 000)	Crude IRR	Adjusted IRR (95 % CI)^Model 1^	Adjusted IRR (95 % CI)^Model 2^
Unexposed	9,225	63.00	1.00(ref)	1.00(ref)	1.00(ref)
Loss of child/spouse	41	107.05	1.67	1.65(1.21–2.24)	1.53(1.13–2.08)
12–7 months	12	93.84	1.46	1.50(0.85–2.65)	1.37(0.78–2.41)
6–0 months	22	112.95	1.76	1.77(1.16–2.68)	1.63(1.07–2.47)
Prenatal	7	116.01	1.81	1.58(0.75–3.32)	1.56(0.74–3.27)
Loss of other relatives	138	64.89	1.03	1.02(0.87–1.21)	1.02(0.86–1.20)
12–7 months	41	68.69	1.10	1.10(0.81–1.48)	1.09(0.80–1.47)
6–0 months	39	65.07	1.02	1.02(0.74–1.39)	1.01(0.74–1.38)
Prenatal	58	62.33	0.99	0.98(0.76–1.27)	0.98(0.76–1.27)

Model 1: Adjusted for country, offspring’s sex, parity, number of fetuses in the pregnancy, paternal age, maternal age, maternal countries of origin, family history of psychiatric disorders, maternal education and calendar year of follow up

Model 2: Adjusted for gestational age and birth weight in addition to the variables from Model 1

*IRR* incidence rate ratio, *CI* confidence intervals

### Sex-difference effects and sensitivity analyses

When stratifying on sex of children, we found an increased risk of ED in children born to mothers who lost a close relative during the six months before conception in boys (IRR: 2.21, 95 % CI: 1.28–3.82), but not in girls (IRR: 1.18, 95 % CI: 0.61–2.27) (Table 5). Results were similar to those from the main analyses after excluding children with a family history of psychiatric disorders (results are available upon request).

**Table 5:** Relative risk of eating disorders and prenatal maternal bereavement stratified on sex of children

Exposure category	Boys			Girls		
	Cases	Incidence rate (1/100,000)	Adjusted IRR (95 % CI)^a^	Cases	Incidence rate (1/100,000)	Adjusted IRR (95 % CI)^a^
Unexposed	4,197	55.84	1.00(ref)	5,027	70.54	1.00(ref)
Any exposed	86	67.10	1.16(0.94–1.43)	93	75.74	1.05(0.86–1.29)
Time of exposure						
12–7 months	26	70.16	1.24(0.85–1.81)	27	76.23	1.05(0.72–1.54)
6–0 months	34	84.30	1.44(1.03–2.02)	27	69.09	0.94(0.65–1.38)
Prenatal	26	51.20	0.88(0.60–1.29)	39	80.76	1.14(0.84–1.56)
1st trimester	10	67.51	1.13(0.61–2.11)	13	91.07	1.24(0.72–2.13)
2nd trimester	9	43.45	0.72(0.38–1.39)	17	85.78	1.23(0.77–1.95)
3rd trimester	7	45.87	0.83(0.40–1.75)	9	63.37	0.92(0.48–1.76)
Type of deceased relative						
Child/spouse	20	103.91	1.69(1.09–2.63)	21	110.22	1.41(0.92–2.16)
12–7 months	4	62.82	1.03(0.39–2.75)	8	124.61	1.76(0.82–3.30)
6–0 months	13	133.11	2.21(1.28–3.82)	9	92.66	1.18(0.61–2.27)
Prenatal	3	96.34	1.45(0.47–4.51)	4	136.99	1.65(0.62–4.39)
Other relatives	66	60.59	1.06(0.83–1.35)	72	69.40	0.98(0.78–1.24)
12–7 months	22	71.68	1.28(0.85–1.93)	19	65.52	0.91(0.58–1.44)
6–0 months	21	68.71	1.19(0.77–1.82)	18	61.29	0.86(0.54–1.36)
Prenatal	23	48.25	0.83(0.55–1.26)	35	77.14	1.11(0.80–1.54)
Cause of death						
Unexpected causes	10	66.31	1.13(0.63–2.05)	11	76.20	0.94(0.52–1.70)
12–7 months	2	44.68	1.04(0.33–3.22)	5	124.50	1.68(0.70–4.04)
6–0 months	4	80.73	1.24(0.47–3.31)	–	–	–
Prenatal	4	70.80	1.06(0.40–2.84)	6	106.33	1.43(0.64–3.18)
Other causes	76	67.52	1.17(0.93–1.46)	82	76.05	1.07(0.86–1.33)
12–7 months	24	73.96	1.26(0.85–1.89)	22	70.38	0.98(0.65–1.50)
6–0 months	30	85.27	1.47(1.03–2.10)	27	79.07	1.09(0.74–1.59)
Prenatal	22	48.96	0.85(0.56–1.29)	33	77.80	1.12(0.80–1.57)

*IRR* incidence rate ratio, *CI* confidence intervals

^a^Poisson regression models were adjusted for country, maternal age, paternal age, parity, number of fetuses in the pregnancy, maternal countries of origin, family history of psychiatric disorders, maternal education, gestational age, birth weight and calendar year of follow up; −. No case

## Discussion

### The main findings of this study

In this large population-based cohort study, we found that maternal exposure to the loss of a core family member (an older child or spouse) the year before or during pregnancy was associated with an increased risk of ED in infants and toddlers. The risk was only significantly increased among children with maternal loss of an older child during six months before conception; whereas it remained high throughout other prenatal periods. The association persisted after excluding children with a family history of psychiatric disorders. When stratifying on sex of children, we only found results similar to the main analyses in boys, but not in girls.

### Comparison with previous studies and potential underlying mechanisms

Research on the effect of prenatal stress on ED in early life has been limited. A case report from 1995 presented a three years old boy with infantile anorexia who experienced prenatal bereavement, and suggested that severe prenatal stress may be implicated in the etiology of ED in infants or toddlers [34]. Two observational studies showed that self-reported maternal anxiety or depression during pregnancy was associated with an increased risk of infant feeding problems [35, 36]. The sample sizes of these studies were substantially smaller than our study and information on confounders was limited. Another study, examining several offspring psychiatric disorders after prenatal (six months before pregnancy and during pregnancy) and postnatal bereavement, showed different vulnerable periods for these diseases [15]. For instance, for ASD, the second and third trimesters were the most vulnerable periods, while only the third trimester was associated with an increased risk for ADHD, and no preconceptional periods were associated with increased risks. We extended the time window to one year prior to pregnancy and observed that only maternal bereavement six months before conception was associated with an increased risk of ED in offspring.

The mechanism of association between maternal stress and risk of disordered behavior in offspring was not well understood. Based on previous studies, the main proposed mechanism for the link between prenatal maternal stress and psychiatric disorders is the programming effect of HPA axis [37]. Postnatal stress is more likely to affect interaction between mother and child in early life of offspring and has a direct effect on children eating behavior [38, 39]. Therefore prenatal stress and postnatal stress might have different mechanisms on the risk of ED. In this study, we focus on prenatal stress’s effect. Our results can be interpreted by increased CRF1 expression in frontal cortex of brain which is related to appetite regulation following maternal preconceptional stress [40, 41]. Class et al. have reported that preconceptional stress increased the risk of infant mortality, which indicated the period immediately before conception may be a sensitive developmental period [42].

It has been suggested that temperament is an important contributor to the development and maintenance of ED [43]. We observed an increased risk of ED but only in boys exposed to maternal bereavement following the loss of a core family member during six months before pregnancy. The result is consistent with suggestions of an animal study, which found that maternal stress before conception may worsen social interaction and increase fear response in male, but not in female rats [44]. In addition, another animal study reported that exposure to stress in the two weeks prior to mating was associated with neuronal morphology change in the anterior cingulate cortex of brain only in males [45], where is on the pathway of appetite activation [46]. Our results suggested that for mothers having male offspring, several months before conception may be a vulnerable time window.

### Strengths and limitations

To our knowledge, this is the first population-based study to explore the association between prenatal stress and the risk of ED in infants and toddlers. Based on nationwide registries of two Nordic countries, we could explore the association between bereavement and ED before the age of 3 with 0.18 % prevalence (rare event). In addition, with almost complete follow-up, the results are unlikely to be biased by selection of study participants or loss to follow up. The large sample size also allowed for adjustment for a number of potentially important confounders. However, our findings should be interpreted with caution due to several limitations. First, we had no data on maternal lifestyle factors, such as prenatal drinking and marijuana use, which might confound the association [47]. For instance, bereaved mothers are likely to drink alcohol, which could partly underlie the association. Second, no systematic study was performed to validate the diagnosis of ED in registries although studies for some major psychiatric diagnoses, such as schizophrenia and single episode depression, have indicated a high validity [48, 49]. Third, patients with mild and moderate ED cannot be identified in the Registries; and the DSM-5 classification (where the feeding and eating disorders have been put together) was not used; and ICD-8 and ICD-10 were used to identify the cases in Denmark while ICD-8, ICD-9 and ICD-10 were used in Sweden. These are expected to lead to bias in disease diagnosis. However, we think that these classifications are independent of exposure status and would thus draw the risk estimates towards unity.

## Conclusions

Our study shows that maternal bereavement related to the loss of a core family member was associated with an increased risk of ED in infants and toddlers. The period of six months before conception may be a susceptible time window, especially for boys.

## References

[1] Treasure J, Claudino AM, Zucker N (2010). Eating disorders. Lancet.

[2] Marchi M, Cohen P (1990). Early childhood eating behaviors and adolescent eating disorders. J Am Acad Child Adolesc Psychiatry.

[3] Chatoor I, Ganiban J, Surles J, Doussard-Roosevelt J (2004). Physiological regulation and infantile anorexia: a pilot study. J Am Acad Child Adolesc Psychiatry.

[4] Manikam R, Perman JA (2000). Pediatric feeding disorders. J Clin Gastroenterol.

[5] Bryant-Waugh R, Markham L, Kreipe RE, Walsh BT (2010). Feeding and eating disorders in childhood. Int J Eat Disord.

[6] Gross MJ, Kahn JP, Laxenaire M, Nicolas JP, Burlet C (1994). Corticotropin-releasing factor and anorexia nervosa: reactions of the hypothalamus-pituitary-adrenal axis to neurotropic stress. Ann Endocrinol.

[7] Licinio J, Wong ML, Gold PW (1996). The hypothalamic-pituitary-adrenal axis in anorexia nervosa. Psychiatry Res.

[8] Gendall KA, Kaye WH, Altemus M, McConaha CW, La Via MC (1999). Leptin, neuropeptide Y, and peptide YY in long-term recovered eating disorder patients. Biol Psychiatry.

[9] Hasan TF, Hasan H (2011). Anorexia nervosa: a unified neurological perspective. Int J Med Sci.

[10] Cummings DE, Frayo RS, Marmonier C, Aubert R, Chapelot D (2004). Plasma ghrelin levels and hunger scores in humans initiating meals voluntarily without time- and food-related cues. Am J Physiol-Endoc M.

[11] Boukouvalas G, Gerozissis K, Kitraki E (2010). Adult Consequences of Post-weaning High Fat Feeding on the Limbic-HPA Axis of Female Rats. Cell Mol Neurobiol.

[12] Glover V, O’Connor TG, O’Donnell K (2010). Prenatal stress and the programming of the HPA axis. Neurosci Biobehav Rev.

[13] Viltart O, Vanbesien-Mailliot CC (2007). Impact of prenatal stress on neuroendocrine programming. TheScientificWorldJOURNAL.

[14] Sominsky L, Spencer SJ (2014). Eating behavior and stress: a pathway to obesity. Front Psychol.

[15] Class QA, Abel KM, Khashan AS, Rickert ME, Dalman C, Larsson H (2014). Offspring psychopathology following preconception, prenatal and postnatal maternal bereavement stress. Psychol Med.

[16] Li J, Olsen J, Vestergaard M, Obel C (2010). Attention-deficit/hyperactivity disorder in the offspring following prenatal maternal bereavement: a nationwide follow-up study in Denmark. Eur Child Adolesc Psychiatry.

[17] Abel KM, Heuvelman HP, Jorgensen L, Magnusson C, Wicks S, Susser E (2014). Severe bereavement stress during the prenatal and childhood periods and risk of psychosis in later life: population based cohort study. BMJ.

[18] Glover V, Hill J (2012). Sex differences in the programming effects of prenatal stress on psychopathology and stress responses: an evolutionary perspective. Physiol Behav.

[19] Ekbom A (2011). The Swedish Multi-generation Register. Methods Mol Biol.

[20] Pedersen CB (2011). The Danish Civil Registration System. Scand J Publ Health.

[21] Mors O, Perto GP, Mortensen PB (2011). The Danish Psychiatric Central Research Register. Scand J Publ Health.

[22] Lynge E, Sandegaard JL, Rebolj M (2011). The Danish National Patient Register. Scand J Publ Health.

[23] Welfare TNBoHa (2009). Kvalitet Och Innehåll i patientregistet.

[24] Chatoor I (2002). Feeding disorders in infants and toddlers: diagnosis and treatment. Child Adolesc Psychiatr Clin N Am.

[25] Axelsson O (2003). The Swedish Medical Birth Register. Acta Obstet Gynecol Scand.

[26] Knudsen LB, Olsen J (1998). The Danish Medical Birth Registry. Dan Med Bull.

[27] Timmermans B. The Danish Integrated Database for Labor Market Research: Towards Demystification for the English Speaking Audience. In*.* Edited by University A. Aalborg University: Aalborg University; 2010.

[28] Li J, Vestergaard M, Obel C, Cnattingus S, Gissler M, Olsen J (2011). Cohort profile: the Nordic Perinatal Bereavement Cohort. Int J Epidemiol.

[29] Skodol AE, Shrout PE (1989). Use of Dsm-Iii Axis-Iv in Clinical-Practice - Rating Etiologically Significant Stressors. Am J Psychiat.

[30] Burton AM, Haley WE, Small BJ (2006). Bereavement after caregiving or unexpected death: effects on elderly spouses. Aging Ment Health.

[31] Lindberg L, Hjern A (2003). Risk factors for anorexia nervosa: a national cohort study. Int J Eat Disord.

[32] Cnattingius S, Hultman CM, Dahl M, Sparen P (1999). Very preterm birth, birth trauma, and the risk of anorexia nervosa among girls. Arch Gen Psychiatry.

[33] Ahren JC, Chiesa F, Koupil I, Magnusson C, Dalman C, Goodman A (2013). We are family--parents, siblings, and eating disorders in a prospective total-population study of 250,000 Swedish males and females. Int J Eat Disord.

[34] Sanchezcardenas M, Mammar N, Venisse JL, Robin D (1995). Complications of Bereavement as Seen in Infant Anorexia and Adolescent Anorexia-Nervosa. Int J Eat Disord.

[35] Farrow C, Blissett J (2006). Maternal cognitions, psychopathologic symptoms, and infant temperament as predictors of early infant feeding problems: a longitudinal study. Int J Eat Disord.

[36] Ammaniti M, Lucarelli L, Cimino S, D’Olimpio F, Chatoor I (2010). Maternal psychopathology and child risk factors in infantile anorexia. Int J Eat Disord.

[37] Weinstock M (2001). Alterations induced by gestational stress in brain morphology and behaviour of the offspring. Prog Neurobiol.

[38] Nicol-Harper R, Harvey AG, Stein A (2007). Interactions between mothers and infants: Impact of maternal anxiety. Infant Behav Dev.

[39] Chatoor I, Egan J, Getson P, Menvielle E, O’Donnell R (1988). Mother-infant interactions in infantile anorexia nervosa. J Am Acad Child Adolesc Psychiatry.

[40] Zaidan H, Leshem M, Gaisler-Salomon I (2013). Prereproductive stress to female rats alters corticotropin releasing factor type 1 expression in ova and behavior and brain corticotropin releasing factor type 1 expression in offspring. Biol Psychiatry.

[41] Pelleymounter MA, Joppa M, Carmouche M, Cullen MJ, Brown B, Murphy B (2000). Role of corticotropin-releasing factor (CRF) receptors in the anorexic syndrome induced by CRF. J Pharmacol Exp Ther.

[42] Class QA, Khashan AS, Lichtenstein P, Langstrom N, D’Onofrio BM (2013). Maternal stress and infant mortality: the importance of the preconception period. Psychol Sci.

[43] Atiye M, Miettunen J, Raevuori-Helkamaa A (2015). A meta-analysis of temperament in eating disorders. Eur Eat Disord Rev.

[44] Shachar-Dadon A, Schulkin J, Leshem M (2009). Adversity Before Conception Will Affect Adult Progeny in Rats. Dev Psychol.

[45] Bock J, Poeschel J, Schindler J, Borner F, Shachar-Dadon A, Ferdman N, et al. Transgenerational sex-specific impact of preconception stress on the development of dendritic spines and dendritic length in the medial prefrontal cortex. Brain Struct Funct. 2014. [Epub ahead of print]. 10.1007/s00429-014-0940-4 25395153

[46] Rolls ET (2006). Brain mechanisms underlying flavour and appetite. Philos Trans R Soc Lond Ser B Biol Sci.

[47] Day NL, Richardson GA, Geva D, Robles N (1994). Alcohol, marijuana, and tobacco: effects of prenatal exposure on offspring growth and morphology at age six. Alcohol Clin Exp Res.

[48] Bock C, Bukh JD, Vinberg M, Gether U, Kessing LV (2009). Validity of the diagnosis of a single depressive episode in a case register. Clin Pract Epidemiol Ment Health.

[49] Loffler W, Hafner H, Fatkenheuer B, Maurer K, Riecher-Rossler A, Lutzhoft J (1994). Validation of Danish case register diagnosis for schizophrenia. Acta Psychiatr Scand.

